# Baicalein Inhibits the SMYD2/RPS7 Signaling Pathway to Inhibit the Occurrence and Metastasis of Lung Cancer

**DOI:** 10.1155/2022/3796218

**Published:** 2022-04-08

**Authors:** Lin Gu, Feng Xu, Xiaoli Zhang, Zhihui Gu

**Affiliations:** ^1^Pharmacy Department, Qingpu Traditional Chinese Medicine Hospital, Shanghai 201700, China; ^2^Pharmacy Department, Shanghai Sixth people's Hospital South Affiliated to Shanghai Jiaotong University, Shanghai 200233, China; ^3^Hospital Director's Office, Qingpu Traditional Chinese Medicine Hospital, Shanghai 201700, China

## Abstract

**Objective:**

This study investigated the potential effects of Baicalein on proliferation, migration, and invasion of human lung cancer A549 and NCI-H1299 cells and its possible mechanisms.

**Methods:**

The effects of Baicalein on proliferation and invasion of A549 and NCI-H1299 cells were detected by MTT assay, clonogenesis assay, and Transwell assay. A subcutaneous transplanted tumor model was used to evaluate the effects of SMYD2 and Baicalein on the proliferation of lung cancer. Baicalein inhibited in SMYD2/RPS7 signaling pathway in tumor cells was also detected by qRT-PCR.

**Results:**

Baicalein significantly inhibited the growth of lung cancer cells. In addition, Baicalein significantly reduced the rate of A549 and NCI-H1299 cell invasion and clone formation in a dose-dependent manner. Animal experiments showed that both SMYD2 and Baicalein treatments could inhibit lung cancer tumor proliferation. Mechanism studies suggest that Baicalein inhibits the SMYD2/RPS7 signaling pathway.

**Conclusion:**

These results indicated that Baicalein could inhibit the proliferation, migration, and invasion of LUNG cancer A549 and NCI-H1299 cells. Baicalein inhibits cell proliferation by downregulating the SMYD2/RPS7 signaling pathway.

## 1. Introduction

Chemotherapy is one of the conventional treatments for lung cancer [1]. However, the side effects of chemotherapy drugs inhibit the immune function of patients and affect their quality of life. Some patients cannot even tolerate it and stop treatment [2]. Because the pathological mechanism of NSCLC is very complex and has not been clearly explained at present, there is still no fundamental treatment for the disease [3].

Baicalein is one of the main flavonoids of Scutellaria baicalensis [4]. Studies have shown that Baicalein has certain antitumor effects and has gradually become a research hotspot [5, 6]. A large number of studies have shown that Baicalein inhibits the invasion and metastasis of malignant tumors through various pathways [7, 8]. Baicalein has been reported to inhibit the growth of laryngeal and esophageal carcinoma. Its mechanism mainly includes influencing arachidonic acid system metabolism, inhibiting tumor cell proliferation, scavenging reactive oxygen species, reversing multidrug resistance of tumor cells, inducing tumor cell apoptosis, inhibiting tumor angiogenesis, and enhancing chemotherapy sensitivity [9, 10]. Most researchers mainly elaborated the antitumor mechanism of Baicalein from the gene and protein levels. Recent studies have suggested novel ideas, including the ability of Baicalein to promote autophagy death of tumor cells. The mechanism is related to the inhibition of AKT/mTOR and AMPK/ULK1 pathways [11–13]. Liu et al. [14] proposed that Baicalein inhibited the of bladder cancer.

SMYD2 is widely distributed in normal, pathological, and tumor tissues [15]. The tumor suppressor gene p53 is the most common inactivated cancer gene in humans. SMYD2 inhibits p53 inhibition activity after monomethylated p53 at lysine residue 370 (p53K370me1) and enhances or inhibits p53 transcriptional activity after methylated p53 at lysine C-terminal [16, 17]. The expression of SMYD2 in esophageal tumor, retinoblastoma, and breast cancer is sensitive and specific. SMYD2 has the tendency of heterologous distribution of tumor [18]. SMYD2 is associated with embryos and various phylogeny and may be a human oncogene. Its overexpression is closely related to malignancy characterization, heterologous distribution, and disease outcome. The sensitivity and specificity of SMYD2 provide a new method for early detection of tumors [19, 20]. Wu et al. reported that SMYD2 promotes the occurrence and metastasis of lung adenocarcinoma through RPS7 [21]. However, the role of SMYD2 in lung cancer remains to be further studied. This study investigated whether the inhibitory effect of Baicalein on lung cancer cells was related to the SMYD2/RPS7 signaling pathway.

In this study, the antitumor effects and mechanisms of Baicalein were analyzed. Meanwhile, the role of SMYD2/RPS7 signaling pathway in lung cancer was analyzed. This study further clarified the material basis and mechanism of Baicalein's antitumor function. This study provides potential targets and lead compounds for the treatment of lung cancer.

## 2. The Materials and Methods

### 2.1. The Tissue and Pathological Information of Lung Cancer Patients Were Collected

Twenty NSCLC patients from the Department of Oncology of our hospital from January 2021 to October 2021 were selected. The following are the inclusion criteria: (1) All patients in the study were confirmed to be NSCLC by pathological section diagnosis. (2) The patients were newly diagnosed and did not receive radiotherapy or chemotherapy, targeted therapy, or immunotherapy before surgery. (3) The general information of the patients was complete, and informed consent had been signed before enrollment. (4) All patients could cooperate with outpatient or telephone follow-up after surgical treatment. The clinical data were as follows: The patient was 58.63 ± 7.23 years old. There were 8 females and 12 males. Pathological specimens of 20 NSCLC patients were selected, and nontumor tissue ≥ 5 cm away from tumor tissue was selected as paracancer tissue. No cancer cells were found in the adjacent tissues by histopathological examination. The study was approved by the Ethics Committee of Qingpu Traditional Chinese Medicine Hospital, and the patients or their families signed the informed agreement.

### 2.2. Bioinformatics Analysis

UALCAN (http://ualcan.path.uab.edu) is a TCGA database website on-line analysis and mining, based on PERL-built CGl, JavaScript, and CSS. This study analyzed the correlation between SMYD7 expression level and prognosis of lung cancer patients through UALCAN website.

### 2.3. Cell Culture

Human non-small-cell lung cancer A549 and NCI-H1299 cells were cultured in RMI1640 complete medium (containing 10% FBS and 1% dual antibody). Culture in saturated humidity, 5%CO_2_, 37°C constant temperature conditions. The solution was changed every other day and digested with trypsin every 3 days. Cells were subcultured in a ratio of 1 : 3, and cells at logarithmic growth stage were selected for experiment. Baicalein was purchased from Sigma-Aldrich (St. Louis, USA). Baicalein (10, 20, and 40 *μ*mol/L) treats cancer cells at the indicated dosages.

### 2.4. Cell Transfection

The cells were counted by trypsin digestion one day before transfection and inoculated with 3.5 × 10^6^ cells per well in 6-well plates for overnight culture. A549 and NCI-H1299 cells were transfected with 45 ng sh-NC and sh-SMYD2, respectively, according to the instructions of Lipofectamine™ 2000 transfection reagent, and cultured at 37°C with 5% CO_2_ for 24 h. Replace fresh medium. The 45 ng pcDNA3.1 empty vector and PCDNA3.1-SMYD2 plasmid were transfected into cells, respectively. The transfected cells were cultured at 37°C and 5% CO_2_ for 24 h; then the fresh medium was replaced. Transfection efficiency was measured at 48 h after transfection. Other experiments were also performed 48 hours after transfection.

### 2.5. QRT-PCR

Take 100 mg tissue sample, and add 1 mL of RNA lysis solution. Shake and digest for 3 to 5 minutes, add about 200 *μ*L of chloroform, and centrifuge at 12,000 g for 15 minutes at 4°C. The supernatant was mixed with 0.5 mL of isopropanol and centrifuged at 12,000 g for 10 min at 4°C. Measure the concentration of RNA after dissolving in enzyme-free water. According to the Takara kit for the configuration of the reaction system, the genomic DNA removal reaction is 42°C and 2 min. The reverse transcription reaction is 37°C, 15 min to 85°C, 5 s. The reverse transcription sample line qPCR reaction system configuration, according to the predenaturation 95°C, 30 s, 40 cycles of amplification. The conditions are set to 95°C (5 s) to 60°C (30 s): GAPDH—5′-GAGTCCACTGGCGTCTTCA-3′ and 5′-GGGGTGCTAAGCAGTTGGT-3′. There are 3 replicate wells in each group, and 2^−*ΔΔ*Ct^ is used to calculate the relative level of target gene mRNA.

### 2.6. Transwell

Coat it with 30 *μ*L diluted Matrigel matrix gel in Transwell cells. Incubate at 37°C for 30 min. The cell density was adjusted to 2 × 10^5^ cells/mL. 5, 10, and 20 *μ*mol/L Baicalein single cell suspension were added into the upper chamber according to grouping conditions. Add 10% serum DMEM medium (600 *μ*L) to the lower chamber. Culture at 37°C with 5% CO_2_ for 24 h. The upper and lower media were removed, fixed with methanol for 10 min, and stained with crystal violet at room temperature for 15 min. Incubate with methanol again for 10 min. Then, the photos were taken under a microscope.

### 2.7. Clone Formation Experiment

The cells at logarithmic growth stage were digested into single cell suspension and placed in a 10 cm dish. Each dish contained 3000 cells. The incubator was placed for 24 h and completely adhered to the wall. After 12 days, the old medium was discarded and cleaned with PBS solution once. The cells were fixed with 4% paraformaldehyde for 15 min. Stain with 0.1% crystal violet for 20 min, wash with PBS solution for 3 times, and take pictures.

### 2.8. Subcutaneous Graft Tumor Model

Twenty-four SPF nude mice aged 4-6 weeks were female, and their body weight was 18-20 g. The mice were provided by Beijing Huafukang Biotechnology Co., Ltd. A549 cell suspension of logarithmic growth phase was inoculated subcutaneously in the right axillary of model mice. Each was 0.1 mL (count after 0.2% Trypanosoma blue staining, the number of living cells > 95%; the concentration was 1 × 10^7^/mL), and the whole process was aseptic. On the 7th day after inoculation, tumor masses were palpable in the right armpit of the model mice. No mice died during modeling. The maximum long diameter (a) and short diameter (b) of the axillary tumor were measured with a vernier caliper every 2 days. Tumor volume was calculated with the mean volume = *A* × *B*^2^/2.

### 2.9. Immunohistochemical Staining

Dewax and hydrate tissue sections. The tissue sections were treated according to the special requirements of the primary antibody used. Incubate with 3%H_2_O_2_ deionized water for 10 min to block endogenous peroxidase. Rinse with PBS, 2 min × 3 times. Primary antibody (dilution concentration: 1 : 100) was dropped and incubated at 37°C for 1-2 h and then washed with PBS, 2 min × 3 times. Polymer Helper was dropped and incubated at 37°C for 20 min. Rinse with PBS, 2 min × 3 times. Add poly-HRP Anti-Rabbit IgG or poly-HRP Anti-mouse IgG. Incubate at 37°C for 20 min. Rinse with PBS, 2 min × 3 times. DAB solution was used for color rendering. Tap water is fully rinsed, redyed, dehydrated transparent, and sealed. Take pictures under a microscope. Image-Pro Plus 6.0 software is used for image processing and analysis.

### 2.10. Detection of Cell Proliferation Ability

The cells were treated into a cell density of 3.0 × 10^4^ cells/mL. Line 100 *μ*L each well into a 96-well plate. The cells were cultured in an incubator for 24 h to make them completely adherent. The cells were treated for 72 h. Add MTT solution in the dark. After incubation at 37°C for 3 h, the MTT solution was sucked out and discarded. Add 150 *μ*L DMSO solution to each well. The absorbance was measured at 490 nm with a microplate reader, and the cell survival rate was calculated.

### 2.11. Statistical Analysis

SPSS 17.0 software was used for statistical processing of data. The data were expressed as the mean ± standard deviation. Comparison between the two groups was performed by a *t*-test. One-way ANOVA was used for comparison between multiple groups, and *P* < 0.05 was considered statistically significant.

## 3. Results

### 3.1. The Relationship between the Expression of SMYD2 and Prognosis in NSCLC Tumor and Paratumor Tissues

**Figure 1:**
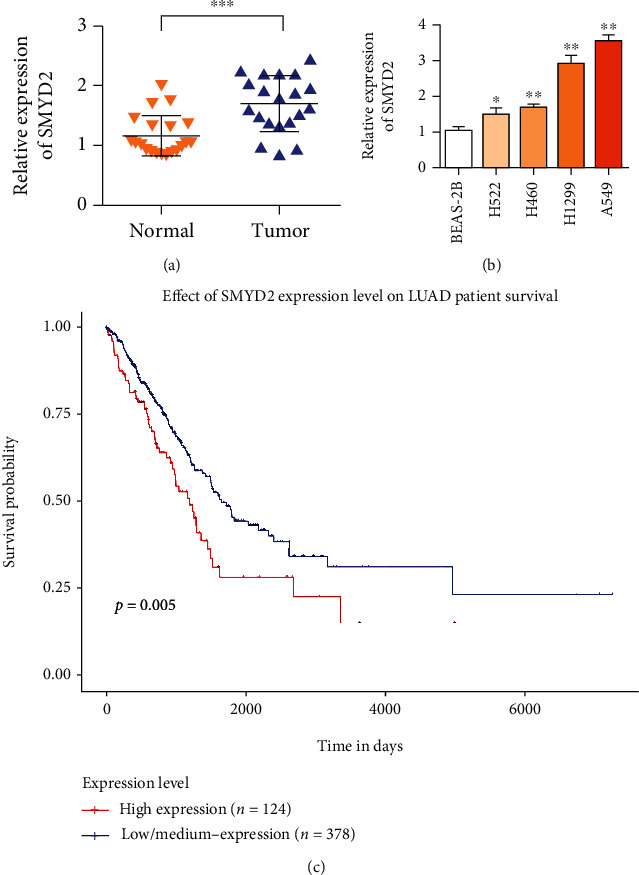
SMYD2 expression is up-regulated in patients with lung cancer. High expression of SMYD2 is associated with poor prognosis of patients. (a) RT-qPCR was used to detect the relative expression of SMYD2 in 20 NSCLC tissues and matched adjacent normal tissues. (b) RT-qPCR was used to analyze the expression level of SMYD2 in NSCLC cell line and normal bronchial epithelial cell line BEAS-2B. (c) High expression level of SMYD2 is associated with poor prognosis of lung cancer (*n* = 502). ^∗^*P* < 0.05 and^∗∗^*P* < 0.01.

SMYD2 expression levels in tumor and paracancer tissues of 20 NSCLC patients were detected and compared. qRT-PCR results showed that the average expression level of SMYD2 mRNA in NSCLC tissues was higher than that in adjacent tissues (Figure 1(a)). In addition, SMYD2 was highly expressed in lung cancer cells A549 and NCI-H1299 (Figure 1(b)). Survival analysis results showed (Figure 1(c)) that the survival curves of patients with positive SMYD2 expression (*n* = 124) are significantly longer than the survival curves of those with negative SMYD2 expression (*n* = 378). These results indicate that the expression level of SMYD2 can be used as an important prognostic indicator of NSCLC patients.

### 3.2. SMYD2 Knockdown Can Inhibit the Proliferation and Invasion of Human Non-Small-Cell Lung Cancer A549 and NCI-H1299 Cells

**Figure 2:**
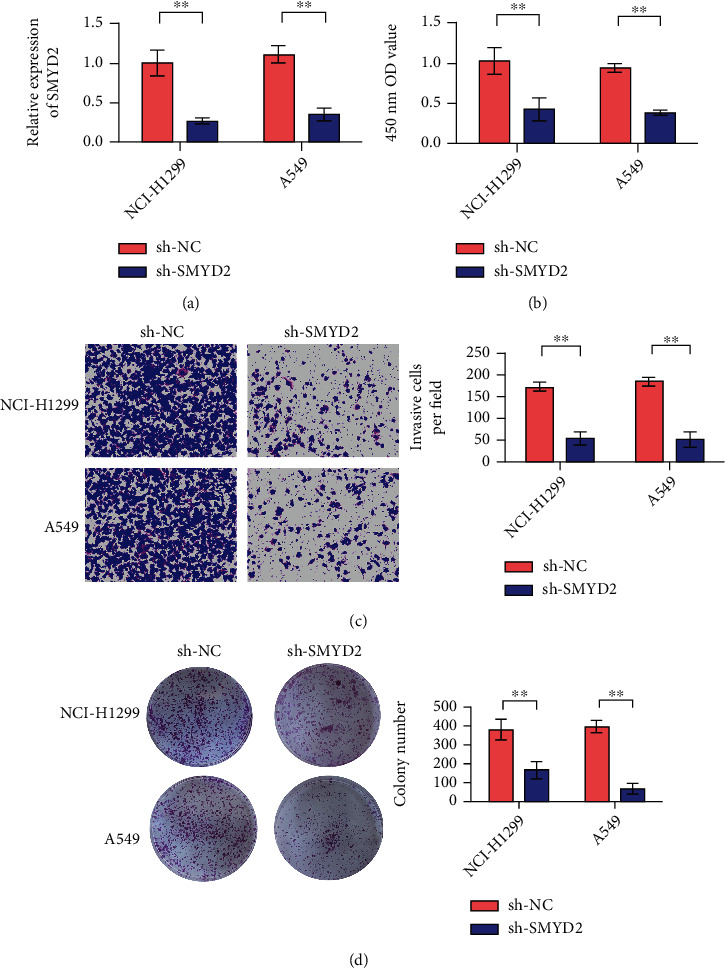
Decreased expression of SMYD2 leads to weakened proliferation, migration and invasion of A549 and NCI-H1299 cells. (a) A549 and NCI-H1299 cells were transfected with sh-SMYD2 and sh-NC, respectively, and SMYD2 levels were detected by RT-qPCR. (b) CCK-8 experiment showed that knocking down SMYD2 inhibited the proliferation of A549 and NCI-H1299 cells. (c) Transwell cell invasion experiment showed that after silencing SMYD2, the invasion ability of A549 and NCI-H1299 cells decreased (×200 times). (d) The clone formation experiment showed that after silencing SMYD2, the cloning ability of A549 and NCI-H1299 cells decreased (×200 times). ^∗∗^*P* < 0.01.

The results in Figure 2(a) showed that sh-SMYD2 could reduce the expression of SMYD2. The MTT method was used to determine the effect of SMYD2 knockdown on the proliferation of human non-small-cell lung cancer cell lines A549 and NCI-H1299, as shown in Figure 2(b). SMYD2 knockdown significantly inhibited the proliferation of A549 and NCI-H1299 cells. Transwell assay results showed that SMYD2 knockdown significantly reduced the invasion ability of A549 and NCI-H1299 cells (Figure 2(c)). The results of clone formation experiment showed that SMYD2 knockdown significantly reduced the clone formation rate of A549 and NCI-H1299 cells, which was statistically significant compared with the control group (Figure 2(d)). In conclusion, SMYD2 knockdown can inhibit the proliferation and invasion of human non-small-cell lung cancer A549 and NCI-H1299 cells.

### 3.3. Knockdown the Effect of SMYD2 on the Growth of Xenograft Tumor in Nude Mice

**Figure 3:**
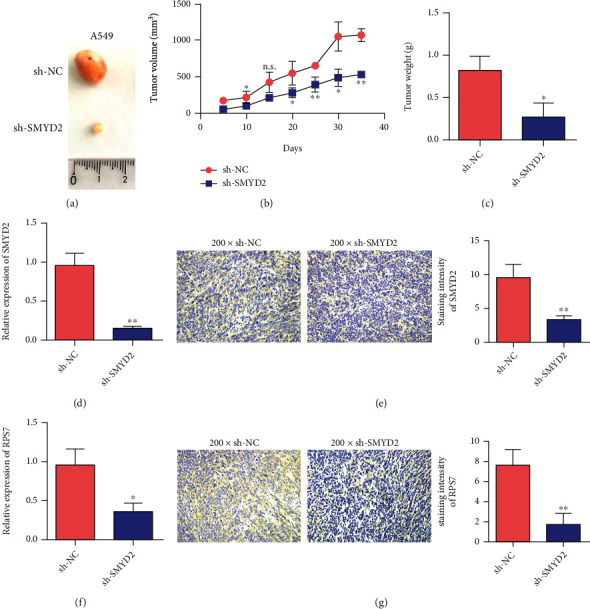
Knockdown of SMYD2 inhibits the growth of lung cancer tumors. (a) Representative pictures of tumors in the control group and the transfection A549 group. (b) Detect the tumor volume of SMYD2 transfected A549 cells. (c) Detect the tumor weight of SMYD2 transfected A549 cells. (d) qRT-PCR detects the expression of SMYD2 in the tumor tissue after SMYD2 transfected A549 cells are tumor-bearing. (e) Immunohistochemical detection of SMYD2 expression in tumor tissues after SMYD2 transfected A549 cells are tumor-bearing. (f) qRT-PCR detects the expression of RPS7 in the tumor tissue after SMYD2 transfected A549 cells are loaded. (g) Immunohistochemical detection of the expression of RPS7 in the tumor tissue after SMYD2 transfected A549 cells are bearing the tumor. ^∗^*P* < 0.05 and^∗∗^*P* < 0.01.

On day 10, the volume of transplanted tumor began to decrease in the SMYD2 knockdown group, but there was no statistical significance. On day 35, the graft volume of SMYD2 group was significantly reduced compared with the control group (Figures 3(a) and 3(b)). Compared with the control group, the weight of transplanted tumor in the SMYD2 knockdown group was smaller than that in the control group (Figures 3(c) and 3(d)). Results showed that SMYD2 expression was decreased in SMYD2 group. Immunohistochemical results showed that the SMYD2/RPS7 signaling pathway was suppressed after SMYD2 knockdown (Figures 3(e)–3(g)).

### 3.4. Effects of Baicalein on the Growth and Invasion of Lung Cancer Cells

**Figure 4:**
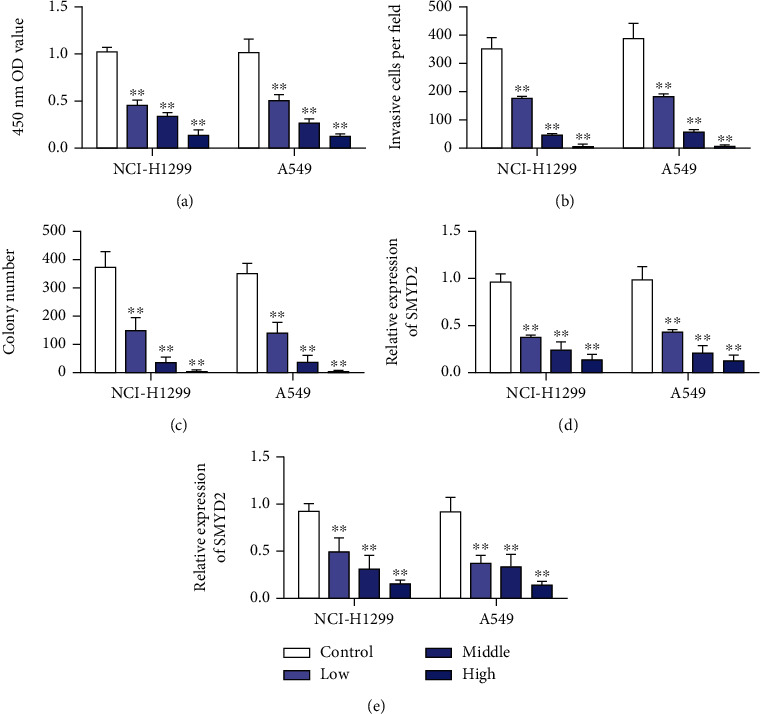
Baicalein targeted inhibition of the SMYD2/RPS7 signaling pathway to inhibit lung cancer cells. (a) Baicalein inhibits the proliferation of lung cancer A549 and NCI-H1299 cell lines in differences dose. (b) Baicalein inhibits the invasion ability of lung cancer A549 and NCI-H1299 cell lines. (c) Baicalein inhibits the cloning ability of lung cancer A549 and NCI-H1299 cell lines. (d) qRT-PCR detection of Baicalein inhibits the expression of SMYD2 in lung cancer A549 and NCI-H1299 cells. (e) qRT-PCR detection of Baicalein inhibits the expression of RPS7 in lung cancer A549 and NCI-H1299 cells. ^∗∗^*P* < 0.01.

Baicalein could inhibit the growth of lung cancer A549 and NCI-H1299 cells in a concentration-dependent manner, and the results showed statistically significant differences (Figure 4(a)). Transwell invasion assay was used to detect the effect of Baicalein on the invasion ability of lung cancer cells. The results showed that Baicalein significantly reduced the invasion of lung cancer A549 and NCI-H1299 cells into the artificial basement membrane. Compared with the control group, there was significant statistical difference (Figure 4(b)). The effect of Baicalein on clonogenesis of lung cancer cells was investigated by clonogenesis assay. The results showed that Baicalein reduced the formation rate of A549 and NCI-H1299 cells in a concentration-dependent manner, which was statistically significant compared with the control group (Figure 4(c)). These results suggest that Baicalein has a strong weakening effect on proliferation and invasion of lung cancer cells. qRT-PCR results showed that the SMYD2/RPS7 signaling pathway was suppressed after treatment with Baicalein (Figures 4(d) and 4(e)).

### 3.5. Baicalein Inhibited the Growth of Transplanted Tumor of Nude Mouse Lung Cancer A549 Cells

**Figure 5:**
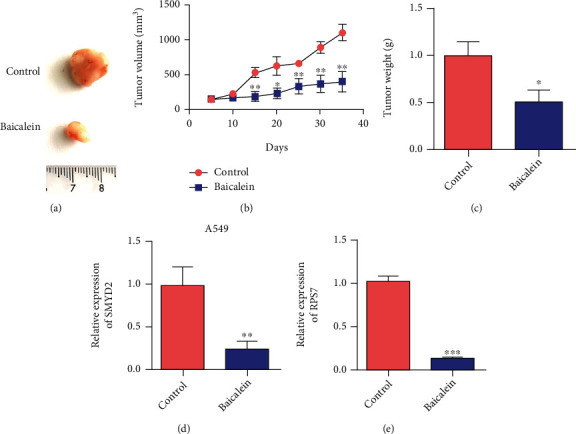
Animal level verification Baicalein inhibits the growth of lung cancer tumors. (a) Representative pictures of tumors in the control group and Baicalein treatment group. (b) Detect the tumor volume of A549 cells after Baicalein administration. (c) Detect the tumor weight of A549 cells after Baicalein administration. (d) Detect the expression of SMYD2 in tumor tissues after administration of Baicalein after A549 cells are tumor-bearing. (e) Detect the expression of RPS7 in tumor tissues after administration of Baicalein after A549 cells are tumor-bearing. ^∗^*P* < 0.05,  ^∗∗^*P* < 0.01, and^∗∗∗^*P* < 0.001.

The results of tumor formation experiment in nude mice showed that the tumor growth of Scutellaria baicalensis group was inhibited compared with the normal saline group. Tumor volume and proliferation rate in the Baicalein group were lower than those in the saline group (Figures 5(a) and 5(b)). Compared with that in the control group, the weight of transplanted tumor in the Baicalein group was smaller than that in the control group (Figure 5(c)). The results of Figures 5(d) and 5(e) showed that the expression levels of SMYD2 and RPS7 were decreased in the Baicalein group. The results showed that Baicalein inhibited the SMYD2/RPS7 signaling pathway.

### 3.6. Baicalein Can Inhibit the Carcinogenic Effect of SMYD2

**Figure 6:**
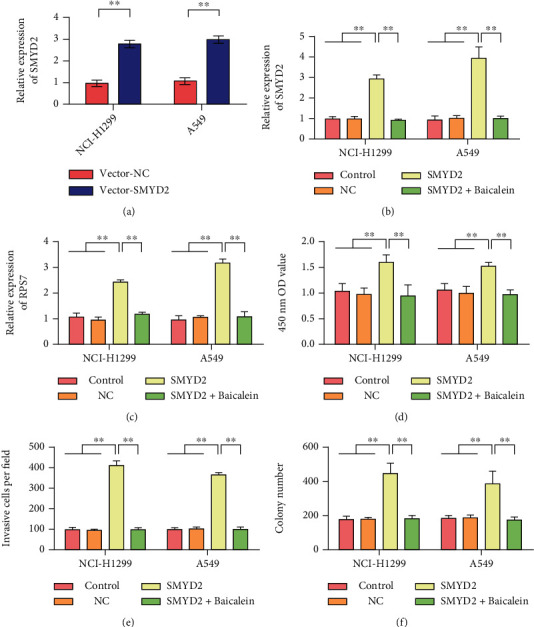
Baicalein inhibits the cancer-promoting effect of SMYD2. (a) Detection of SMYD2 overexpression efficiency in A549 and NCI-H1299 cells. (b) After different treatments, detection of SMYD2 expression in A549 and NCI-H1299 cells. (c) After different treatments, the expression of RPS7 in A549 and NCI-H1299 cells was detected. (d) After different treatments, the proliferation rate of A549 and NCI-H1299 cells was detected. (e) Detection of invasion ability of A549 and NCI-H1299 cells after different treatments. (f) After different treatments, the clone formation ability of A549 and NCI-H1299 cells was tested. ^∗∗^*P* < 0.01.

A549 and NCI-H1299 cells were transfected with SMYD2 overexpressed plasmid, and transfection efficiency was measured. The results of Figure 6(a) showed that the expression of SMYD2 in A549 and NCI-H1299 cells was significantly increased after transfection (Figure 6(a)). The results of Figures 6(b) and 6(c) showed that the expression levels of SMYD2 and RPS7 in cells were upregulated after overexpression of SMYD2. Baicalein treatment significantly inhibited the expression of SMYD2 and RPS7 in A549 and NCI-H1299 cells. Cell proliferation experiment results showed that overexpression of SMYD2 promoted proliferation of A549 and NCI-H1299 cells (Figure 6(d)). Baicalein treatment could inhibit the proliferation of SMYD2. Transwell and clonogenesis assays showed that overexpression of SMYD2 promoted the invasion and clonogenesis of A549 and NCI-H1299 cells. In contrast, treatment with Baicalein inhibited the carcinogenic effect of SMYD2 (Figures 6(e) and 6(f)).

## 4. Discussion

With the increase of lung cancer incidence, it is of great significance to the research of lung cancer drugs [22]. Due to the advantages of multitarget, low toxicity, and low cost, more and more attention has been paid to the antitumor effect of traditional Chinese medicine [23]. Currently, Baicalein has been reported to have some antitumor effects [24]. However, its specific mechanism of action is not very clear and needs to be further elucidated. Inhibiting the proliferation and migration of tumor cells can inhibit the growth of tumor [25]. In this study, Baicalein significantly inhibited the proliferation of human non-small-cell lung cancer cells A549 and NCI-H1299. Tumor cell metastasis is an important cause of high mortality of cancer. In this study, Baicalein inhibited the in vitro migration of A549 and NCI-H1299 cells. Moreover, it could inhibit the invasion process of NCIH1299 cells to a certain extent. MTT assay and clonogenesis assay showed that Baicalein could inhibit of A549 cells in a concentration and time-dependent manner. The mechanism of Baicalein against proliferation, migration, and invasion of lung cancer cells was preliminarily explored in this study. It was found that Baicalein could inhibit the proliferation, invasion, and metastasis of lung cancer cells by inhibiting the expression of SMYD2 in A549 cells.

Studies have shown that Baicalein has antitumor effects. Baicalein has certain anticancer effects on various cancers such as oral cancer, breast cancer, and bladder cancer [10, 26]. Baicalein works by regulating certain pathways that inhibit oral cancer cells. Chung et al. [27] found that Baicalein inhibits TGF-B1-mediated EMT through NF-*κ*B pathway expression level. And in subsequently increased migration, Baicalein reduced TGF-B1-mediated EMT in human breast cancer cells (MDA-MB-231 cells). Wang et al. [28] found that Baicalein mainly mediated cell cycle arrest in G0/G1 phase within 12 h of treatment of breast cancer cells. After 12 h, breast cancer cells mainly stagnated in S phase. Baicalein also inhibits bladder cancer cell viability and induces apoptosis. Yang et al. [29] used Baicalein to target mRNA and protein expression corresponding to antiapoptotic genes in T24 and 253J bladder cancer cells. RT-PCR showed that antiapoptotic genes were significantly overexpressed in bladder cancer cells.

Epigenetic modification abnormalities are widely present in the occurrence and development of tumors. Among them, histone methylation modification is the focus of research. Histone methylation modification enzymes are involved in regulating a variety of cellular biological functions, such as heterochromatin formation, X chromosome inactivation, transcriptional regulation, stem cell maintenance, and differentiation [30]. A large number of studies have shown that histone methylation modification enzymes can affect the transcription of oncogenes and tumor suppressor genes or modify the function of proteins. Histone methylation modification enzymes can promote the cascade amplification and interactive regulation of abnormal cell signals, affecting malignant transformation of cells [31]. SMYD contains the SET domain and MYND domain proteins; SMYD is an important group of lysine methyltransferases. Among them, studies on the methylation sites and functional properties regulated by SMYD2 in tumors are extensive, and SMYD2 mainly plays a carcinogenic role [32]. SMYD2 promotes lysine methylation of MAPKAPK3 at site 355 in pancreatic ductal adenocarcinoma [33]. SMYD2 has been found to promote RAS-mediated pancreatic cancer formation in in vitro cell and animal studies. Interference with SMYD2 expression enhances chemotherapy sensitivity of tumor cells [34]. In non-small-cell lung cancer cells with emL4-ALK gene fusion, inhibition of SMYD2 expression or protein catalytic function leads to decreased levels of methylation and phosphorylation of EML4-AlK protein K1610, which inhibits cell growth [35]. In addition, SMYD2 can promote the proliferation, migration and invasion of gastric cancer, esophageal squamous cell carcinoma, head and neck tumor cells, and other malignant biological functions [19, 36, 37]. In this study, SMYD2 expression was upregulated in lung cancer patients. High expression of SMYD2 is associated with poor prognosis. Reduced expression of SMYD2 resulted in reduced proliferation, migration, and invasion of A549 and NCI-H1299 cells. In addition, animal experiments showed that SMYD2 knockdown inhibited lung cancer tumor growth.

The results showed that Baicalein could inhibit the proliferation, migration and invasion of lung cancer cells. These results suggest that Baicalein has good antitumor potential, which is worthy of continuous attention and further study.

## 5. Conclusion

In conclusion, Baicalein can inhibit the proliferation and migration of human non-small-cell lung cancer cells. Signaling pathway regulation studies showed that Baicalein also inhibited the SMYD2/RPS7 signaling pathway in human non-small-cell lung cancer cells. This work provides a new content for the study of Baicalein against lung cancer. Further, Baicalein provides a new theoretical basis for clinical treatment of lung cancer. This study has certain reference significance for further study on the antitumor mechanism of Baicalein. It also provides potential therapeutic targets for lung cancer.

## Data Availability

The analyzed datasets generated during the study are available from the corresponding author on reasonable request.
